# The Ultrastructural Changes of the Sertoli and Leydig Cells Following Streptozotocin Induced Diabetes 

**Published:** 2012

**Authors:** Davoud Kianifard, Rajab Ali Sadrkhanlou, Shapour Hasanzadeh

**Affiliations:** 1 *Department of Basic Sciences, Faculty of Veterinary Medicine, University of Tabriz, Tabriz, Iran *; 2 *Department of Basic Sciences, Histology and Embryology Sections, Faculty of Veterinary Medicine, Urmia University, Urmia, Iran*

**Keywords:** Diabetes, Electron Microscopy, Leydig cell, Sertoli cell, Testis

## Abstract

**Objective(s):**

This investigation was conducted to evaluate the effects of diabetes on the structure and function of testicular tissue.

**Materials and Methods:**

Diabetes was induced in male adult rats by a single intraperitoneal injection of streptozotocin. Body and testicular weight, hormonal analyses, histological and ultrastructural analyses were measured.

**Results:**

The body and testicular weights were dropped significantly (*P*< 0.05) in diabetic rats in comparison with control rats. On the other hand, in diabetic rats, the blood glucose level increased significantly (*P*< 0.05). The blood plasma levels of testosterone, 17-β estradiol, progesterone, FSH and LH were reduced in diabetic rats. Histomorphological studies were revealed reduction in diameter of seminiferous tubules and germinal epithelium height, edema in interstitial tissue, germ cell depletion, decrease in cellular population and activity with disruption of spermatogenesis in diabetic rats. Ultrastructural study showed the mitochondrial change and reduction of smooth endoplasmic reticulum in Sertoli and presence of lipid droplets in Leydig cells of diabetic rat’s testes.

**Conclusion:**

The results of the present study confirmed that, the ultrastructural changes of Sertoli and Leydig cells, brought about by streptozotocin** i**nduced diabetes, because of the alterations in pituitary gonadotropins, and these changes influence the normal spermatogenesis in rats.

## Introduction

The complications of diabetes are the major problems that occur in diabetic patients (1). Increase of blood glucose levels leads to structural and functional changes in various target tissues and organs (2). Experimentally induced diabetes in male rats is associated with alteration in functions of reproductive system (3). Streptozotocin (STZ)-induction of diabetes in rats has been adapted as a model for studying and evaluating the effects of diabetes on various organs (4, 5). In addition, the STZ does not have a direct effect on morphological changes in testicular tissue of STZ-induced diabetic rats (6, 7). It has been reported that, insulin insufficiency and the impairment of regulatory action of this hormone on Leydig and Sertoli cells have a major role in testicular alterations of diabetic patients (6). The endocrine functions of testes are control by the gonadotropins of pituitary gland (6). Follicle-stimulating Hormone (FSH) regulates the functions of Sertoli cells which are required for normal spermatogenesis and the luteinizing hormone (LH) controls the Leydig cells functions (8). Reduction of insulin secretion in STZ-induced diabetic rats alters the spermatogenesis through the change in serum FSH levels. Moreover, the relationship between insulin and LH has been demonstrated and according to these findings insulin regulates serum LH levels (6). As the most important hormone for regulation of glucose metabolism, insulin has an imperative role on the control of cell proliferation and metabolism of Leydig cells. These observations indicate that, the regulation of testicular function is the result of multiple mechanisms that include the combined effects of insulin/glucose, FSH, and LH. The effects of diabetes on the microscopic structure of testicular tissue have been reported. In this regard, degeneration of germ cells, giant cell formation, interstitial tissue changes, reduction in the size of seminiferous tubules and the changes in Leydig cells were demonstrated (9, 10). Moreover, the effect of diabetes on the changes of body and testicular weights has been reported in the previous studies (1, 2, 11, 12). Gonadal dysfunction and decrease in testosterone production are the consequence of diabetes and these conditions lead to insufficient production of spermatozoids (10, 13, 14).

There are limited data about the effects of ultrastructural changes of Sertoli and Leydig cells (as the target cells of pituitary gonadotropins) on the normal functions of testes and spermatogenesis after induction of diabetes; therefore, this study was designed to find the relationships between the changes of blood glucose level with the alterations of the blood level of gonadotropins and testicular steroids and the association between these hormonal changes with the histological and ultrastructural remarks of testicular tissue in adult male diabetic rats.

## Materials and Methods

***Animal procedure***

Adult male Sprague-Dawley rats with a body weight 200±20 g were used in this study. They were provided by the Center of Animal Housing and Breeding of the Faculty of Veterinary Medicine of Urmia University. The baby rats were placed in standard cages (2 animals per each) under light for 12-hour: dark cycle with room temperature of 23 -25˚C until they reached the desired body weight for the study to begin. All animals received standard laboratory animal's chow and water *ad labitum* during the whole period of experiment. In this study, all animal procedures were carried out in accordance with the standards for human care and use of laboratory animals which has been approved by animal welfare. 

***Experimental design***

In this study, the animals were divided into two different experimental groups of 8 in each group: (1) control: normal and apparently healthy rats that did not receive any type of treatment; (2) diabetic: in the animals of this group the experimental diabetes was induced by a single intraperitoneal injection of STZ with dose of 45 mg/kg of body weight. To the animals of control group, in state of streptozotocin, same volume of citrate buffer was injected intraperitoneally. The duration of all experiment was 10 weeks after induction of diabetes.

***Treatments and chemicals***

In this study, the streptozotocin (Sigma, ST. Louis, MO, USA) was used for induction of diabetes in rats. The STZ dissolved in 0.1 M citrate sodium buffer (pH 4.5) and was injected to overnight fasting animals. Diabetes was confirmed 48 hours after injection of STZ. For this aim, the blood glucose levels of fasting animals was collected from tail vein and determined with automated glucose analyzer device (Glucometer, On Call EZ, SD, USA). The animals with blood glucose levels above 200 mg/dl were considered diabetic and were used in this study (15). 

***Weighing of body and gonads, and blood collecting***

At the end of the study, the weight of each animal was recorded. Then the overnight fasting rats were anesthetized with diethyl ether. For measurement of plasma glucose levels and hormonal analyses, the blood was immediately collected by cardiac puncture and plasma was separated from the blood cells by centrifugation. Then, all blood plasma samples were immediately stored in -20 ˚C for further analyses. After weighing and sacrificing of animals, the right and left gonads of each rat were separated from the body and their weights recorded**.**

***Analytic procedures in plasma samples***

The blood glucose levels were determined by spectrophotometery according to the glucose-oxidase method (Unico 1200, Japan), blood plasma testosterone, 17-β estradiol and progesterone levels were measured by an enzyme-linked immunosorbent assay (ELISA) method using a commercial kit (Diaplus Inc. USA), blood plasma FSH and LH levels were determined by an ELISA assays using a specific commercial kits (DRG Instruments GmbH, Germany).

***Tissue preparation and histological techniques***

The testicular tissues were immediately fixed in 10% formaldehyde in buffered solution containing 54 mM NaH_2_PO_4_ and 28 mM Na_2_HPO_4_ (pH 7.4) and kept at 4˚C. After 48 hours, the transverse section was made on the middle part of each testis and kept immersed in the fixative solution for the completion of tissue fixation. Then, formaldehyde-fixed samples were embedded in paraffin and sliced with thickness of 6-7 micrometer and were mounted onto albumin-precoated glass slides. The mounted tissue samples were deparaffined with xylol and stained by the Hematoxylin and Eosin method for histological observations by light microscopy.

***Morphometeric analysis***

For morphometeric assessment of seminiferous tubules, the slides were studied at x200 magnification. To get extra precise results, the seminiferous tubules that sectioned transversely were studied and one of them with the shortest diameter was considered for measurement. The analyses were performed from images obtained and digitalized using an Olympus DP70 digital camera (Olympus Europe, Hamburg, Germany). Then, the images were processed by the computerized image analysis system software cell^*^ (Olympus Soft Imaging Solutions GmbH, Münster, Germany). The scale bar was 200 µm and twenty tubules from each specimen were measured in different fields of tissue.

***Evaluation of spermatogenesis in testicular tissue***

Forestimation of spermatogenesis in testicular tissue, three different indices were used. Tubular differentiation index (TDI), repopulation index (RI) and spermiogenesis index (SPI). To determine the tubular differentiation index, the number of seminiferous tubules with more than three layers of germinal cells derived from type A of spermatogonia was calculated. To find out the repopulation and spermiogenesis indices, the ratio of active spermatogonia to inactive cells and the ratio of the number of seminiferous tubules with spermatozoids to the empty tubules, respectively were calculated (16).

***Tissue processing for electron microscopy***

Immediately after collection of blood, the animals were sacrificed by overdose of diethyl ether. For electron microscopy, each testis was separated from its adjacent epididymis and cut into small pieces and fixed in 4% glutaraldehyde in a phosphate buffer (pH 7.2) for 2-4 hrs and post-fixed in 1% osmium tetraoxide. The samples subsequently were dehydrated in graded alcohols and embedded in Spurr resin embedding medium. Semithin and ultrathin sections were obtained using ultramicrotome (LEICA ULTRACUT R, Austria). After monitoring, semithin sections (approximately 1 µm) was stained with toluidine blue, the ultrathin sections were mounted on copper grids and stained with uranyl acetate and lead citrate and observed with a Philips C-100 Bio transmission electron microscope at 80 kV.

***Statistical analyses***

Results were analyzed using the SPSS version 18. All data were reported as Mean ± SEM. To evaluate the significant differences, the comparison of scores between experimental groups was done by computer program for student t test. Differences were considered to be statistically significant if *P*< 0.05.

## Results

***Effects of diabetes on body and gonadal weights***

The mean body weight of animals in experimental groups is shown in Table 1. The body weight of animals in diabetic group was significantly (*P*< 0.05) decreased in comparison to untreated healthy rats at the end of study. As well, the weight of right and left testes in STZ-induced diabetic rats, was decreased significantly (*P*< 0.05) in comparison to control group.

***Effects of diabetes on blood glucose and testicular steroids***

**Figure 1:**
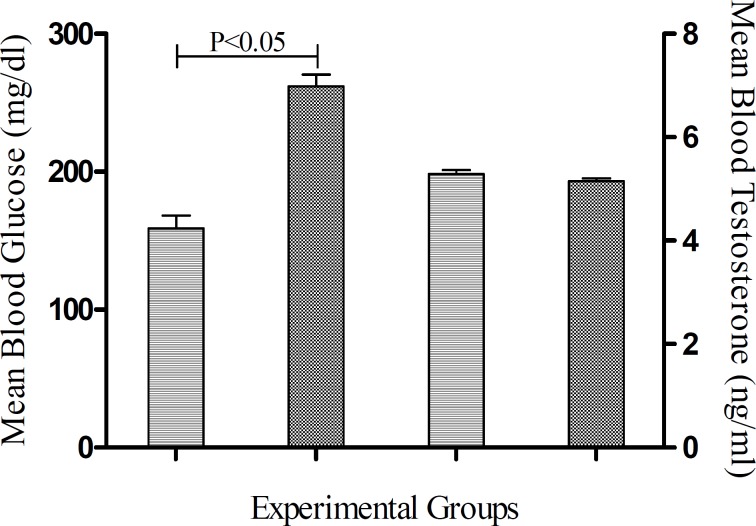
Blood glucose and testosterone levels at the end of study. Data columns are mean ± SEM. Capped line indicates statistically significant difference between two groups

**Figure 2:**
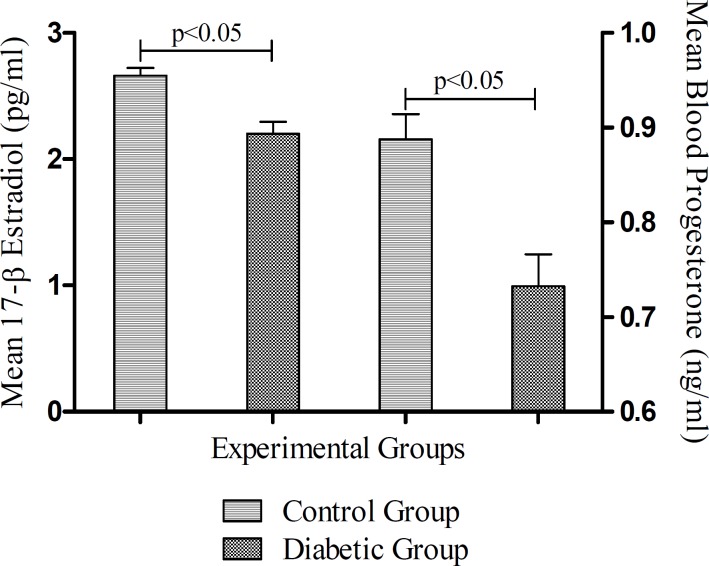
Blood 17-β estradiol and progesterone levels at the end of study. Data columns are mean ± SEM. pped line indicates statistically significant difference between two groups

The mean level of glucose in STZ-induced diabetic rats was significantly higher than untreated control group (*P*< 0.05) (Figure 1). The blood testosterone levels in STZ-induced diabetic rats were decreased in comparison to untreated control rats (Figure 1), however this decrement was not significant. As the Figure 2 illustrates, the mean blood levels of 17-β estradiol and progesterone in STZ-induced diabetic rats are significantly lower than control group (*P* < 0.05).

**Table 1:** The change of body and gonad weights after induction of diabetes (mean± SE

groups		*Body Weight (g)*	*Right gonad weight (g)*	*Left gonad weight (g)*
Control		257.5±4.902	2.360±0.110	2.275±0.045
Diabetic		160.0±8.976^*^	1.659±0.134^*^	1.663±0.152^*^

In each column the asterisk indicates significant difference (*P*< 0.05) between untreated control and STZ-induced diabetic rats.

**Table 2:** Morphometeric analyses of seminiferous tubules in experimental groups after induction of diabetes (mean± SE).

groups	*S T Diameter (µm)*	*G E Height (µm)*	*S T Lumen diameter (µm)*
Control	467.304±7.852	148.121±4.214	171.061±8.238
Diabetic	394.015±26.319^*^	108.521±8.846^*^	176.937±11.803

In each column the asterisk indicates significant difference* (P*< 0.05) between untreated control and STZ-induced diabetic rats. ST designates seminiferous tubule; GE designates germinal epithelium.

***Effects of diabetes on blood gonadotropins levels***

**Figure 3:**
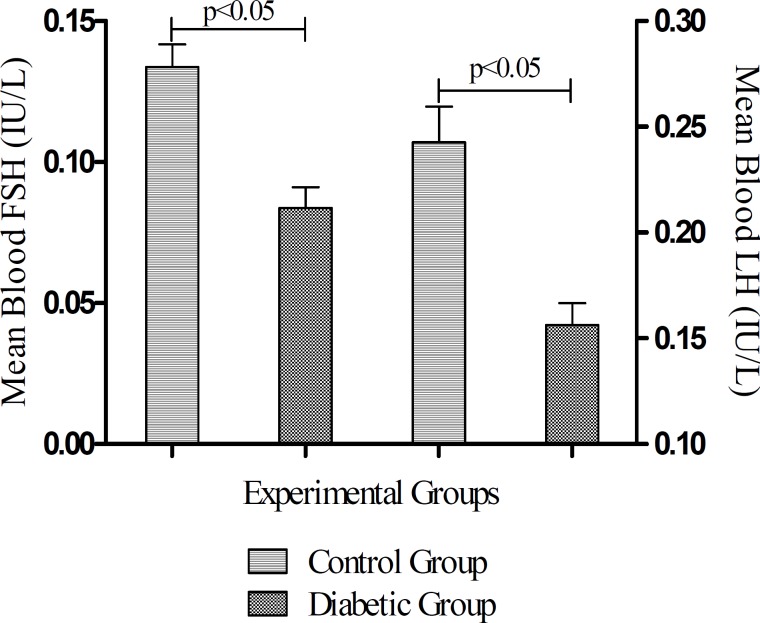
Blood FSH and LH levels at the end of study. Data columns are mean ± SEM. Capped lines indicate statistically significant difference between two groups

The levels of FSH reduced significantly (*P*< 0.05) in STZ-induced diabetic rats in comparison with Control group (Figure 3). There was also, a significant (*P*< 0.05) reduction in the mean of LH level in the STZ-induced diabetic group in comparison to the untreated control rats. 

***Morphometeric analyses of testicular tissue***

The histomorphometeric study of seminiferous tubules (STs) demonstrated that, the diameter of these tubules decreased in diabetic rats. This reduction was significant (*P*< 0.05) between STZ-induced diabetic and control rats (Table 2). These results were satisfactory about the germinal epithelium as well, the height of germinal epithelium of STs decreased significantly (*P*< 0.05) in diabetic group in comparison with control rats. 

The width of the lumen of seminiferous tubules somewhat increased (was not significant) in diabetic rats in comparison with control group. 

***Effects of diabetes on the epithelium of seminiferous tubules***

The quantitative analysis of the cells of seminiferous tubules revealed that, in the STZ- induced diabetic rats the number of Sertoli cells significantly (*P* < 0.05) decreased in comparison to control group (Table 3). Moreover, in STZ-induced diabetic rats the number of spermatogonia cells was decreased in comparison to other group however, this difference was not significant. As Table 3 demonstrates, the number of primary spermatocytes and round spermatids were reduced after development of diabetes and this reduction was significant between diabetic and control rats (*P*< 0.05). 

**Table 3:** Effects of streptozotocin induction of diabetes on the cellular population of germinal epithelium of seminiferous tubules (Mean±SEM).

groups	*Sertoli cells*	*Spermatogonia*	*Primary spermatocytes*	*Spermatids*
Control	17.66 ± 1.115	60 ± 2.0816	60.33 ± 2.3475	176.66 ± 11.4183
Diabetic	13.50 ± 1.5220^*^	44.83 ± 5.7062	51 ± 2.1291^*^	149.50 ± 11.9798^*^

In each column the asterisk indicates significant difference (*P*< 0.05) between untreated control and STZ-induced diabetic rats.

**Table 4:** Effects of streptozotocin induction of diabetes on spermatogenesis indices(Mean±SEM).

groups	*T D I (%)*	*S P I (%)*	*R I (%)*
Control	90.6412 ± 1.9382	88.6903 ± 0.8404	82.7449 ± 2.9411
Diabetic	79.8396 ± 1.9863^*^	72.6835 ± 4.1050^*^	60.5072 ± 3.2252^*^

In each column the asterisk indicates significant difference (*P*< 0.05) between untreated control and STZ-induced diabetic rats.

***Effects of diabetes on spermatogenesis***

Table 4 demonstrates the three indices determined for spermatogenesis. All the indices of spermatogenesis were reduced significantly (*P*< 0.05) in diabetic group in comparison to control group.

***Histological observations of testicular tissue***

**Figure 4:**
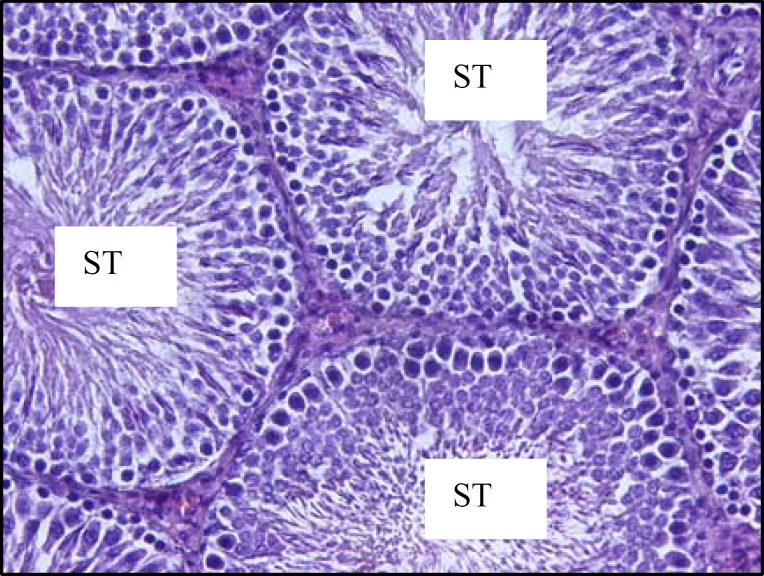
Light micrograph of testicular tissue of a rat belongs to the control group. The seminiferous tubules (ST) have ordinary shape; their epithelium is structurally intact and shows normal association of germ cells. H&E staining method, (×200).

**Figure 5:**
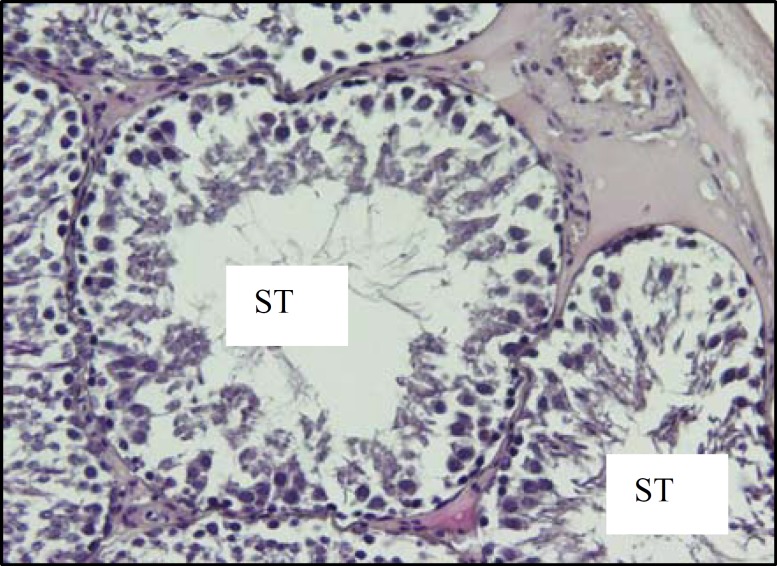
Light micrograph of testicular tissue of a rat belongs to the control group. The seminiferous tubules (ST) have irregular shape and the germinal epithelium is disorganized. Depletion of germ cells is obvious. H&E staining method. (×200).

**Figure6:**
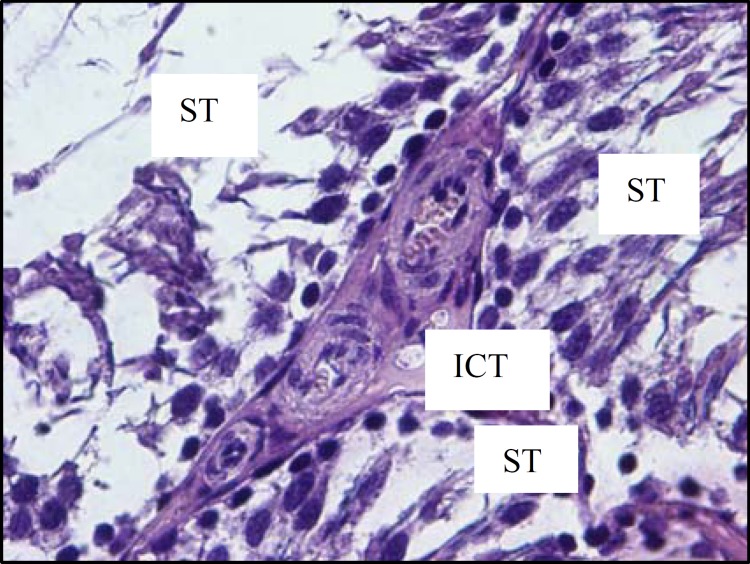
Cross section showing part of three seminiferous tubules (ST) of a rat from diabetic group. The edema was attenuated in interstitial connective tissue (ICT). H&E staining method, (×400)

**Figure 7:**
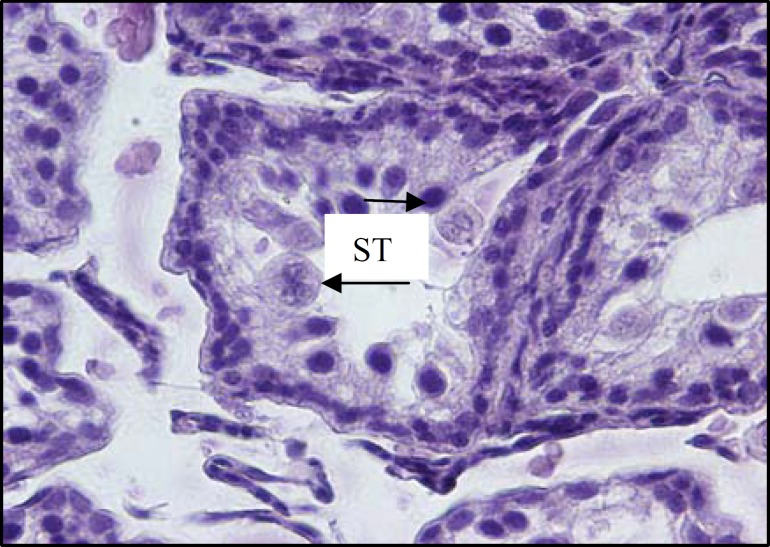
Light micrograph showing part of testicular tissue from an animal rendered diabetic. The giant cell formation with two or three nucleus (arrows) is seen in the lumen of irregular shaped seminiferous tubule (ST). H&E staining method, (×400).

The histological investigations of testicular tissue in different groups demonstrated that, in diabetic rats the seminiferous tubules were irregular in shape, the normal organization of germinal epithelium was reduced and the cells of germinal epithelium had abnormal cellular attachment, in addition, some extent of depletion in spermatogenic cells was seen. As well, the round spermatids had some malorientation and the number of cell layers was reduced (negative TDI) (Figures 5, 6). In testicular tissue of control rats, the STs had compacted and organized germinal cells and all types of cells had normal cellular attachment, moreover, five or more cell layers were seen in the epithelium of seminiferous tubules (positive TDI) (Figure 4). In diabetic rats the interstitial connective tissue had the amorphous material and the edema was attenuated in interstitial connective tissue (Figure 6). The multinucleated cells with two or three nucleus were revealed in some of seminiferous tubules in diabetic cases whereas, this type of cells was not observed in control group (Figure 7).

**Figure 8:**
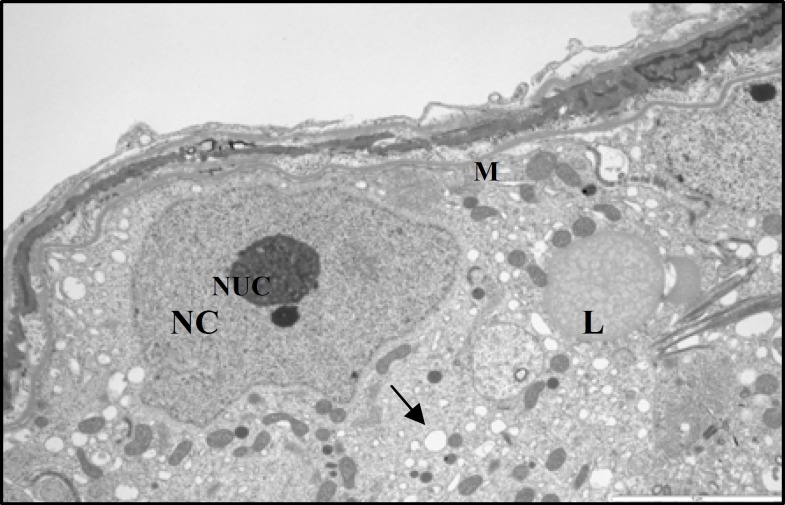
Electron micrograph showing part of seminiferous tubule from a control rat. The normal Sertoli cell with large nucleus (NC) and its nucleolus (NUC). Note a heterochromatin clump is in close association with nucleolus. The cytoplasm filled with numerous circular mitochondria (M) and smooth endoplasmic reticulum (arrow). A lipid granule (L) also seen in the cytoplasm (× 5800) .

***Ultrastructural study***

**Figure 9:**
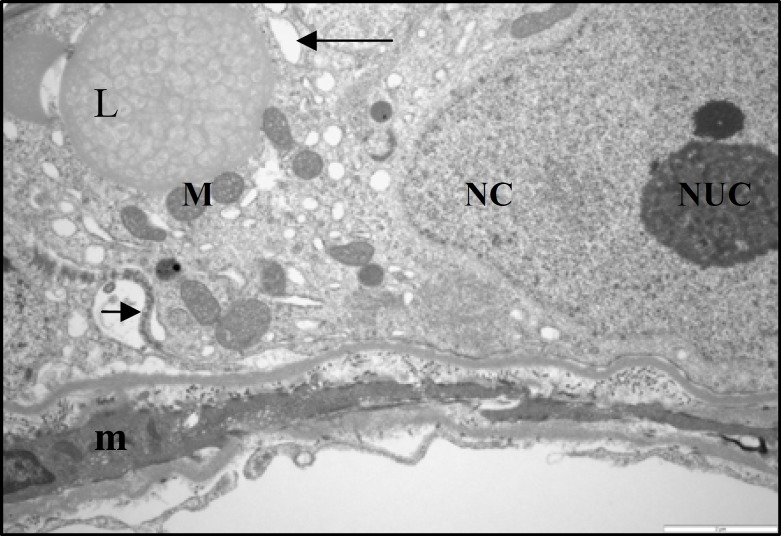
Electron micrograph showing part of seminiferous tubule from a control rat with higher magnification. The normal sertoli cell with large nucleus (NC) and its nucleolus (NUC) resting perpendicular to the basal lamina (*). The part of cytoplasm with circular mitochondria (M) and smooth endoplasmic reticulum (arrow) is seen. A lipid granule (L) also seen in the cytoplasm. Sertoli-Sertoli junctional complex seen between adjacent sertoli cells (arrow head). A myoid cell (m) with its cytoplasm located below the basal lamina also seen, (× 9700).

**Figure 10:**
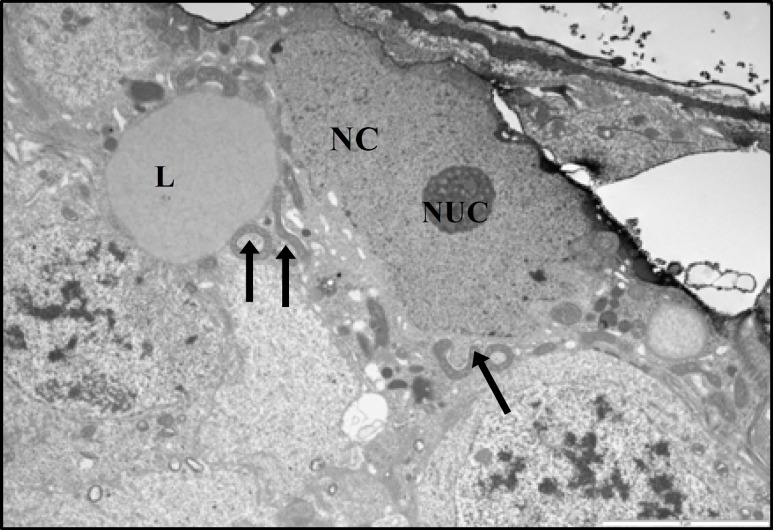
Electron micrograph from part of seminiferous tubule of diabetic rat showing sertoli cell. The cell has irregular nucleus (NC) with nucleolus (NUC). The lipid granule (L) becomes larger. The mitochondria transformed to screwed and/or doughnut-shaped pattern (arrows), (× 5800)

**Figure 11:**
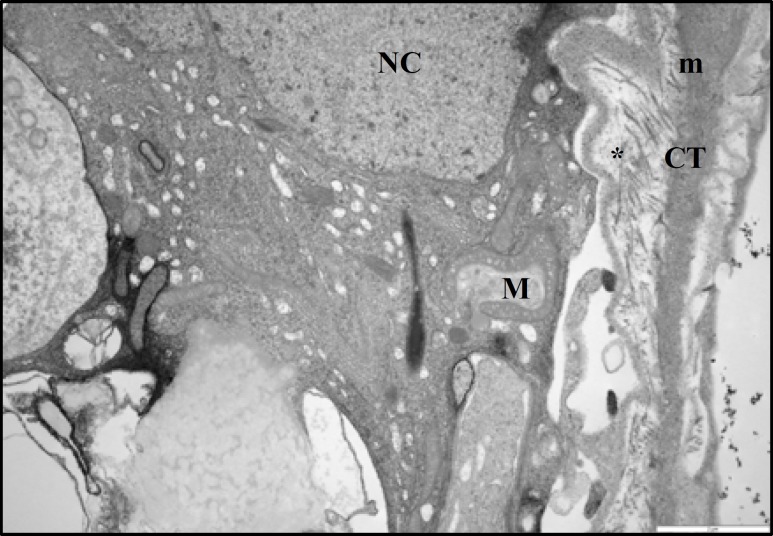
Electron micrograph from part of sertoli cell of testis from a diabetic rat. Note the basal lamina (*) becomes more irregular and the condense collagen fibers (CF) are seen between basal lamina and cytoplasm of myoid cell (m), (× 9700).

At ultrastructural levels, the seminiferous tubules of control rats showed a different cell types, occupying variable proportions of the epithelium. The basal portion of Sertoli cells rest on the basement membrane and the apical region extended to the lumen of seminiferous tubule. The oval-shaped nuclei of the Sertoli cells were readily visible and situated close to the basement membrane. The nucleolus was prominent in the most various sections of the Sertoli cells and the electron-dense perinuclear heterochromatin clumps also were seen (Figures 8, 9). Specialized intercellular tight junctions that cover the areas of contact between the adjacent Sertoli cells were present. In this area the obliterated intercellular spaces were seen. These spaces belong to dilated cisterns of the endoplasmic reticulum settled between the dense filamentous band of junctional complex and the cytoplasm of the Sertoli cell on both sides (Figure 9). The cytoplasm of Sertoli cells contained abundant smooth endoplasmic reticulum and numerous mitochondria. The mitochondria of Sertoli cells had a circular outline with electron-dense matrix and incomplete cristae (vesicular type mitochondria); cup-shaped mitochondria located close to the endoplasmic reticulum, was also observed (Figures 8, 9). The myoid cells with spindle-shaped nuclei and delicate cytoplsamic processes were located around the wall of STs. The space between the basement membrane of STs and myoid cells filled with collagen fibers (Figure 9). The ultrastructural study of Sertoli cells in STZ-induced diabetic rats revealed that, almost all Sertoli cells had remarkable alterations in their fine structure. The oval-shape nuclei became irregular and some abnormalities were seen in cytoplasmic organelles including mitochondria and endoplasmic reticulum (Figure 10). The most remarkable changes in cellular organelles were observed in mitochondria. Normally formed mitochondria of the vesicular type were transformed to giant organelles with irregular cristae. These organelles reduced in number and were arranged in screwed and/or doughnut-shaped pattern (Figures 10, 11). In close conjunction with formation of giant mitochondria, the large lipid vesicles were seen (Figures 10, 11). The smooth endoplasmic reticulum was reduced and the basement membrane of the Sertoli cells showed more irregularity. Moreover, the thickness of the wall of seminiferous tubules was increased. The myiod cells were separated from the STs by more collagen fibers (Figure 11). There was no remarkable abnormality in the junctional specializations between adjacent Sertoli cells. Near the lumen of seminiferous tubule, cytoplasmic fragments of Sertoli cells were observed with dense content. They contained a large number of vacuoles and mitochondria with an ill-defined internal structure.

**Figure 12:**
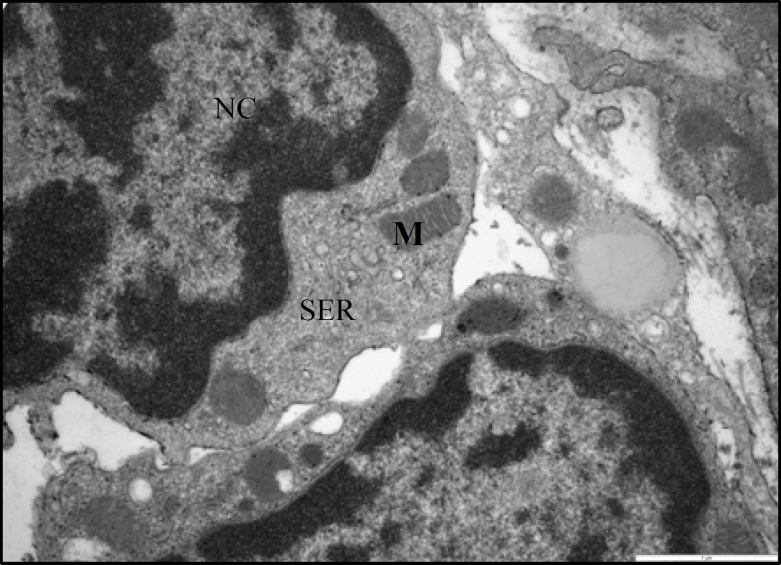
Electron micrograph showing part of two leydig cells from a normal rat. Extensive arrays of smooth endoplasmic reticulum (SER) and normal tubular form of mitochondria (M) are seen in the cytoplasm. Clumps of heterochromatin located in marginal part of nucleus (NC), (× 24500).

**Figure 13:**
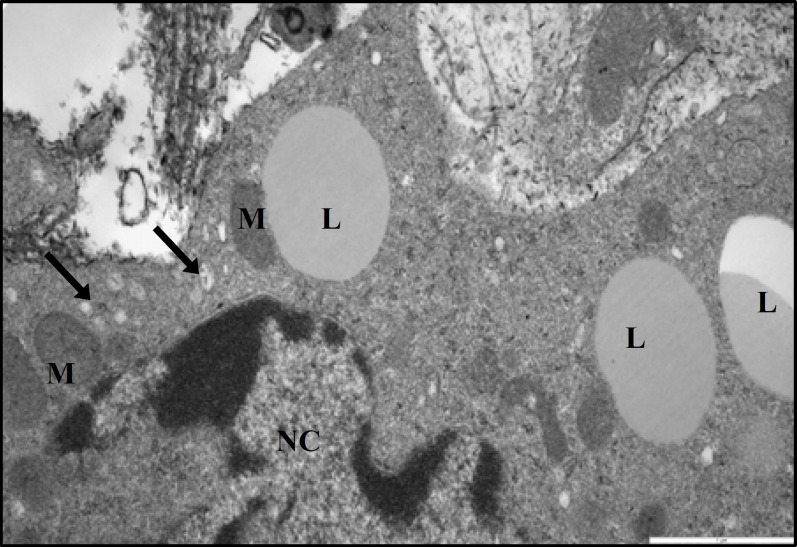
Electron micrograph showing part of leydig cell from a diabetic rat. Note the presence of lipid droplets (L) in association of mitochondria (M). The smooth endoplasmic reticulum is sparse and appears in most parts near the periphery of cell, (×24500).

The ultrastructural study of Leydig cells from normal control rats showed that, they appeared essentially identical to interstitial cells of rats previously described (3). They had an apparently normal complement of organelles such as numerous mitochondria with lamellar cristae, extensive smooth endoplasmic reticulum that was found in many steroid-secreting cells and prominent rim of heterochromatin beneath the nuclear membrane (Figure 12). Ten weeks after induction of diabetes, ultrastructural study of testicular interstitial tissue of STZ-induced diabetic rats revealed polymorphic Leydig cells with irregular nuclei with greater amounts of heterochromatin (Figure 13). The Leydig cells exhibited a number of fine structural alterations in cytoplasmic organelles such as a marked depletion of smooth endoplasmic reticulum. This organelle appeared as poorly organized patches near the marginal regions of the cells (Figure 13). Lipid droplets were present in the majority of the Leydig cells (Figure 13). Mitochondria in Leydig cells of diabetic rats showed normal structure in most respects.

## Discussion

Increase in blood glucose level leads to structural and functional changes in target organs of diabetic patients (2). In our study, the mean blood glucose level of diabetic rats significantly increased above the normal level, and this elevation in blood glucose levels was approximately constant through the course of diabetes. Reduction in pancreatic β-cell mass is associated with development of diabetes (14). In this study, streptozotocin was used for induction of diabetes. This alkylating agent causes pancreatic β-cell death. 

Our experiment revealed that, the mean body weight of diabetic rats significantly reduces during the course of diabetes. The reduction of body weight can be due to the breakdown of tissue proteins in diabetic rats (1, 17). In our study, a reduction of the weight of gonads was seen in diabetic group, this reduction of gonadal weight may happen due to testicular atrophy and/or reduction of the weight of epididymides (not measured) in diabetic rats. The effects of experimental diabetes on the functions of reproductive system and disturbances of hypothalamus-pituitary-gonadal axis have been reported (10, 18, and 19). Diabetes can provoke a reduction in biosynthesis and metabolism of androgens and in streptozotocin induced diabetic rats, this reduction of testicular androgen synthesis have been reported in several studies (4, 10, and 20). The reduction in androgen production can be related to reduction of blood gonadotropins levels (4, 21-23). The mechanism of reduction in production of testosterone might be a direct effect of glucose or its metabolites on gonadotropins or its resistance to these hormones. Moreover, a recent study demonstrated that, there is a direct correlation between blood testosterone and gonadotropins levels (12). It is reported that, induction of diabetes by streptozotocin lead to decrease of blood testosterone levels in diabetic rats in comparison with healthy normal rats. Whereas, some other studies reported, there is no change in blood testosterone levels in such diabetic rats (24-26). In our study, the mean blood testosterone level in diabetic rats decreased in comparison with untreated healthy rats, but this decrement was not significant. These controversies in results of such studies may be related to different coinciding parameters such as strains of rats and/or methods of studies. 

In STZ induced diabetic rats the conversion of steroid precursors to androgens upsets, therefore, the turnover rates of pregnenolone and progesterone markedly decrease accordingly in these animals, and the ability of Leydig cells for metabolization of progesterone to testosterone reduce markedly within three weeks after induction of diabetes. It has been suggested that, estrogens have a possible key role in the augment of complications in testicular endocrinal functions in diabetic animals (4). In our study, the mean blood levels of 17-β estradiol and progesterone decreased in diabetic rats. Decreased levels of 17-β estradiol and progesterone in blood plasma may be due to accumulation of these hormones in testicular tissue of diabetic rats (4). Our results indicate that, there is a close relationship between the blood plasma levels of testosterone with progesterone and estradiol levels, thus the change of blood glucose levels may exert significant role in alterations of these testicular hormones.

The results of this study revealed that, the mean blood level of FSH in diabetic rats experiencing a significant reduction in comparison to control rats. According to results of a study lack of insulin in STZ-induced diabetic rats can affect the serum FSH levels (6). The FSH acts synergistically with the LH in stimulation of androgen synthesis therefore, the reduction of this gonadotropin can play an important role in decrement of testosterone output in diabetic animals (3). 

One of the most important functions of insulin is the modulation of blood FSH levels and the strong correlation have been found between FSH and insulin levels in blood plasma (27, 28). Diabetes induces a reduction of blood plasma LH levels, which is responsible for normal function of Leydig cells (29). The effect of insulin on Leydig cells is related to control of the cell proliferation and metabolism (6). In our study, the blood LH levels significantly lowered in diabetic rats. Insulin has essential role in maintenance of LH receptors on Leydig cells (3). The relationship between LH and insulin has been shown in transgenic mice that lacked brain insulin receptors. A significant reduction in number of Leydig cells was reported in this type of animals (6). It is reported that, the number of LH binding sites in Leydig cells from diabetic rats severely lowered after induction of diabetes (10). These processes lead to depression of synthesis and secretion of testosterone by Leydig cells. The reduction of FSH and LH lead to apoptosis in germinal cells that have testosterone receptors (2). These findings indicate the important role of insulin and/or glucose in regulation of the pituitary gland's function in synthesis and secretion of FSH and LH subsequently, for normal functions of gonads in production of sexual hormones. 

The abnormal spermatogenesis in diabetic conditions was reported in several studies (30, 31).According to our results, in diabetic rats the spermatogenesis is disrupting in comparison with control rats. Decrease in diameter of STs has been reported in previous studies (2, 11). In our study, decrement in diameter of STs was accompanied with depletion in the height of germinal epithelium. This finding indicates that, the STs became atrophied during the course of diabetes. These histological observations in STs, illustrate depressed cellular activity of spermatogenic cells. A reduction in the population of the cells of STs confirms these histological changes. In this study, the number of spermatogonia cells decreased in diabetic testes however, this reduction statistically was not significant. In addition, the number of primary spermatocytes decreased significantly in comparison with control rats. This finding indicates that, the conversion of spermatogonia to primary spermatocytes is reduced in diabetic conditions. Diminished tubular differentiation and spermiation indices in diabetic rats confirm this change in cellular activities of seminiferous tubules. The reduction of repopulation index in diabetic rats demonstrates that, the number of inactive spermatogonia increased after induction of diabetes. This process cause a reduction of the number of primary spermatocytes derived from spermatogonia cells. These alterations in cellular conversion and/or activity lead to reduction in production of spermatozoids. It has been reported that, the expansion of interstitial regions in testicular tissue in diabetic rats take place (32). In our study, this condition was observed. This change of interstitial connective tissue is associated with microvascular angiopathy in diabetic conditions (33). In this study, the histological changes of testicular tissue are comparable and in accordance with the studies that reported the degeneration and necrosis of STs, giant cell formation and interstitial tissue changes (34-36). 

The ultrastructural study of testicular tissue in diabetic rats demonstrated remarkable changes in Sertoli and Leydig cells. In Sertoli cells, the most prominent changes were observed in mitochondria. These organelles had some abnormalities in their shapes and quantity. These changes in structure of mitochondria may reflect the abnormal function of theses organelles. Insulin resistance is the most important factor in pathogenesis of diabetes. Morphological and functional studies have been provided for an intrinsic mitochondrial defect in insulin resistance conditions (37). It has been reported that, in patients with type 2 diabetes, the activities of some enzymes in mitochondria decreases and also some abnormalities in the shape of these organelles have been noted in relation to diabetic conditions (37). Low concentrations of glucose in *ad luminal* parts of STs have an effect on the metabolism of the cells of this region of STs. It has been reported that, lactate is the better energy source for spermatocytes and spermatids (38). The production of lactate from glucose has been done in Sertoli cell and the FSH has an important role in facilitating of glucose transport. Since some abnormalities occur in metabolism of glucose in diabetic conditions, it is believed that, the Sertoli cells have to create the energy sources from other metabolic pathways and these factors may have an important role in the structural changes of organelles involving the metabolism of cells. Sertoli cells are responsible for the control and normal functions of the testicular tissue (32). The germ cells of seminiferous tubule are spermatogonia, spermatocytes, spermatids, and the mature spermatozoa. The process of germ cell development (spermatogenesis) is mainly supported by the Sertoli cells. Therefore, the regulation of Sertoli cell function is one of the most critical essentials influencing spermatogenesis, testis function, and male reproduction. The critical regulatory steps within the testis that influence Sertoli cells and the process of spermatogenesis are cell–cell interactions between the various testicular cell types. 

It was recognized that germ cells can influence the function of Sertoli cells. For instance, the spermatids appear to be particularly essential regulators of Sertoli cells (32). Specific anatomical structures exist between spermatids and Sertoli cells. They are essential mediators in the interaction between these cells. It has been reported that, spermatids play an important role in gene expression and secretary activities of Sertoli cells. In early stages, spermatids, affect the Sertoli cells through the secretion of soluble factors whereas, the effect of late spermatids on Sertoli cells secretion may be mediated via their implication in conformational changes on Sertoli cells (39). The increase of thickness of the wall of STs appears to be caused by increased collagen content. This increase in fibrous part of the wall previously reported (3, 13) and may represent peritubular fibroblasts’ dysfunction (40). A marked depletion of Smooth endoplasmic reticulum in both Sertoli and Leydig cells may reflect an involution of this cellular organelle, which were involved in steroid hormone production. The Leydig cells of normal adult rats do not contain lipid droplets (3) therefore; in our study the presence of lipid droplets in Leydig cells of diabetic rats could represent an accumulation of steroid precursors. 

## Conclusion

The results of this study reveal that, the ultrastructural changes of Sertoli and Leydig cells brought about by streptozotocin induced diabetes cause the alterations in pituitary gonadotropins, and these changes influence the normal spermatogenesis in rats. These changes influence the normal organization and function of spermatogenic cells, thus the alteration in populations of germinal epithelial cells of seminiferous tubules arise after induction of diabetes.
